# Schisandrin Attenuates Lipopolysaccharide-Induced Lung Injury by Regulating TLR-4 and Akt/FoxO1 Signaling Pathways

**DOI:** 10.3389/fphys.2018.01104

**Published:** 2018-08-08

**Authors:** Kai Sun, Rong Huang, Li Yan, Dan-Tong Li, Yu-Ying Liu, Xiao-Hong Wei, Yuan-Chen Cui, Chun-Shui Pan, Jing-Yu Fan, Xian Wang, Jing-Yan Han

**Affiliations:** ^1^Department of Physiology and Pathophysiology, School of Basic Medical Sciences, Peking University, Beijing, China; ^2^Department of Integration of Chinese and Western Medicine, School of Basic Medical Sciences, Peking University, Beijing, China; ^3^Tasly Microcirculation Research Center, Peking University Health Science Center, Beijing, China

**Keywords:** leukocyte, TLR-4, FoxO1, adhesion molecules, tight junction

## Abstract

**Objective:** Acute lung injury is a severe clinic condition with limited therapeutic approaches. This study evaluated whether schisandrin (Sch), an ingredient of *Schisandra chinensis*, has preventive effects on endothelium and epithelium injury induced by lipopolysaccharide (LPS) and the underlying mechanisms.

**Methods:** Male Wistar rats were continuously infused with LPS (5 mg/kg/h) via the left jugular vein for 90 min. In some rats, Sch (2.5 mg/kg/h) was administrated through the left jugular vein 30 min before LPS infusion. Leukocyte recruitment, levels of inflammatory cytokines, lung histology and edema, vascular and alveolar barrier disruption and related proteins were evaluated at indicated time point after LPS challenge.

**Results:** LPS infusion for 90 min resulted in an increased leukocyte adhesion to pulmonary venules and overproduction of cytokine and chemokine in both serum and lung homogenate. At 8 h after termination of LPS infusion, obvious Evans blue extravasation and lung edema were observed, along with an increased apoptosis, a decreased expression of tight junction and adherent junction proteins, and a reduction in von Willebrand factor (vWF) and keratin, all of which were attenuated by Sch treatment. Meanwhile, the LPS-elicited activation of TLR-4/NF-κB/MAPK and FoxO1 signaling was inhibited by Sch.

**Conclusion:** The present study revealed that pretreatment with Sch alleviated lung endothelium and epithelium injury after LPS stimulation, which is attributable to inhibition of cell injury and activation of cell regeneration via regulation of TLR-4/NF-κB/MAPK and Akt/FoxO1 signaling pathway.

## Introduction

Acute lung injury (ALI) and its more severe form, acute respiratory distress syndrome (ARDS) are severe condition in clinic with high mortality and morbidity (Rubenfeld et al., 2005; Herridge et al., 2011; Bellani et al., 2016). ALI occurs as a consequence of a range of insults, including Gram-negative sepsis, which, however, follows a pathophysiological process independent of etiology. Two phases are recognized in ALI progression: acute and resolution. In acute phase, ALI manifests inflammation and resultant disruption of endothelial and epithelial barrier, leading to alveolar flooding with protein rich fluid. Successive repair of damaged endothelial and epithelial cells, clearance of lung edema and removal of proteins in alveolar space are required for ALI progression to resolution phase, and thus are critical for the outcome of the patients (Matthay et al., 2012).

The fate of damaged endothelium and epithelium depends on both the protection of the cell injury and activation of cell regeneration. The cell death is the consequence of the impact of pro-inflammatory cytokines and oxidative stress, while cell regeneration occurs through activation of intrinsic repair programs (Zhao et al., 2006; Mirza et al., 2010). Studies showed that forkhead box O (FoxO), one subfamily of the fork head transcription factor family, plays important roles in cell fate decisions. The FoxO family confers a cell-cycle arrest, pro-apoptotic phenotype on cells (Eijkelenboom and Burgering, 2013). *In vivo* myocyte-specific transgenic expression of FoxO1 during heart development causes embryonic lethality because of severe myocardial defects and decreased myocyte proliferation (Evans-Anderson et al., 2008). Another study shown that genetic ablation of FoxO1 in the liver rescues hepatocyte proliferation, glucose homeostasis, and survival during liver regeneration *in vivo*, and pharmacological inhibition of FoxO1 in mouse primary hepatocytes stimulates the expression of proliferative markers (Pauta et al., 2016). A recent study has shown that FoxO1 could antagonize FoxM1-dependent endothelial regeneration and vascular repair in LPS-induced ALI (Huang et al., 2016). In addition, FoxO1 is involved in the maintenance of pubertal mammary stem cell, in which the inhibition of FoxO1 protected stem cell from excessive apoptosis (Lv et al., 2017; Sreekumar et al., 2017). These data suggest regulating FoxO1 function as a promising strategy for tissue repair.

**FIGURE 1:**
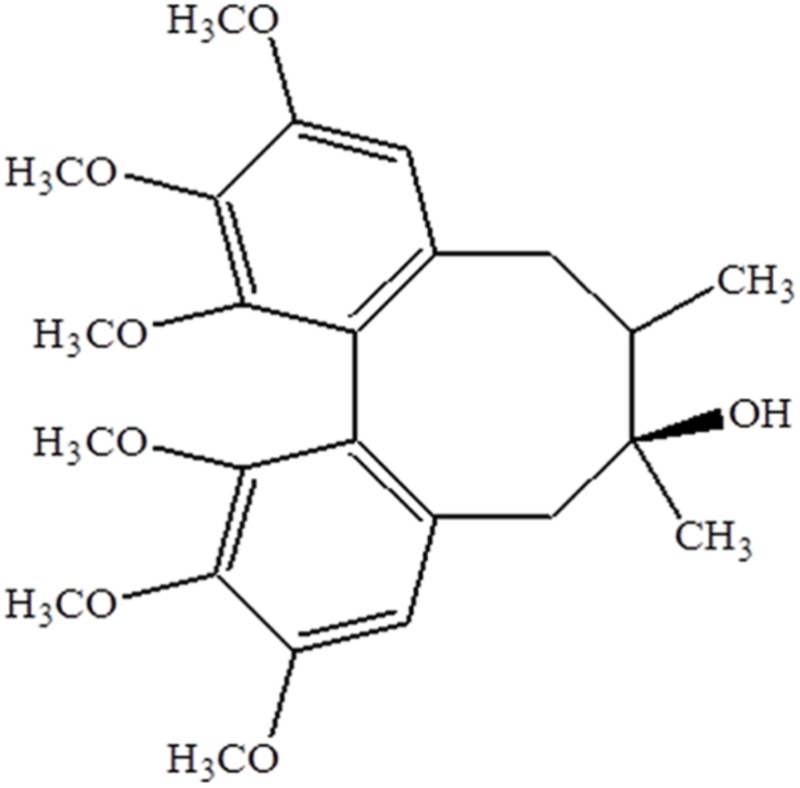
The chemical structure of Sch.

Schisandrin (Sch, **Figure 1**) is a major active ingredient of *Schisandra chinensis* (SC), an extensively used traditional medicine in Asian countries and Russia (Panossian and Wikman, 2008). Sch has been reported to have a variety of pharmacological activity, including anti-inflammation (Lee et al., 2010), anti-oxidation (Jeong et al., 2012), anti-apoptosis (Cheng et al., 2008), anti-allergic reaction (Lee et al., 2007), etc. A previous study has shown that Sch has a protective effect on LPS-induced sepsis increasing the animal survival rate. Sch significantly inhibited carrageenan-induced paw edema and acetic acid-induced vascular permeability in mice, which was associated to inhibiting the activation of NF-κB and MAPK signaling pathway (Guo et al., 2008). Our previous study has demonstrated that pretreatment with Yiqifumai injection (a traditional Chinese medicine preparation containing SC as a major component) attenuates LPS-induced microcirculatory disturbances and hyperpermeability in mesenteric microvessels (Yuan et al., 2009). In the present study, using LPS-induced rat ALI model, we investigated whether Sch has protective effects on endothelium and epithelium injury in lung and the underlying mechanisms.

## Materials and Methods

### Animals

Male Wistar rats (200 ± 20 g) were obtained from the Animal Center of Peking University Health Science Center (Certificate code SCXK 2006-0008). The animals were housed in cages at 22 ± 2°C and humidity of 40 ± 5% in a 12-h light/dark cycle, and received standard diet and water *ad libitum*. The experimental procedures were carried out in accordance with the European commission guidelines (2010/63/EU). All animals were handled as per the guidelines of the Peking University Animal Research Committee, and the surgical procedures and experimental protocol were approved by the Committee on the Ethics of Animal Experiments of the Health Science Center of Peking University (LA2011-38).

### Reagents

Chromatographically pure Sch was purchased from Feng-Shan-Jian Medicine Research Co. Ltd. (Kunming, Yunnan, China) (Structure shown in **Figure 1**), and dissolved in 0.5% dimethyl sulfoxide (DMSO) at a concentration of 0.5 mg/mL before experiment. LPS (*Escherichia coli* serotype O55:B5), Evans blue (EB) dye, and rhodamine 6G were purchased from Sigma Chemical (St. Louis, MO, United States). Primary antibodies against I-κBα, phospho-I-κBα, NF-κB p65, Erk1/2, phospho-Erk1/2, p38 MAPK, phospho-p38 MAPK, phospho-FoxO1, Akt, phospho-Akt, Keratin, caspase-3, cyclin D1, cyclin B1, p27^kip1^, phospho-retinoblastoma protein (Rb), GAPDH, and histone H3 were obtained from Cell Signaling Technology (Beverly, MA, United States). Antibodies against Claudin-5, Occludin and ZO-1 were from Invitrogen (Rochford, IL, United States), antibodies against FoxO1, p21^cip1^, myeloperoxidase (MPO) and von Willebrand factor (vWF) from Abcam (Cambridge, United Kingdom). Antibody against VE-Cadherin from Santa Cruz Biotechnology (Santa Cruz, CA, United States), antibody against TLR4 from Novus Biologicals (Littleton, CO, United States) and antibody against prosurfactant protein C (proSP-C) from EMD Millipore Corporation (Temecula, CA, United States).

### Animals Treatment and Grouping

The experiment was divided into two parts. In the first set of experiment, rats were randomly divided into seven groups (see **Table 1** for details). After being anesthetized with pentobarbital sodium (60 mg/kg, i.p.), the rat left femoral vein and jugular vein were cannulated, as previously described (Zhang et al., 2014). In the LPS group, LPS solution in saline was infused (5 mg/kg/h) for 90 min via the left femoral vein, while only saline was administrated in Sham and Sch alone groups. In the Sch pretreatment group, Sch solution was infused continuously through the left jugular vein 30 min before LPS administration at the dose of 0.625 mg/kg/h, 1.25 mg/kg/h, 2.5 mg/kg/h or 5 mg/kg/h. Saline and Sch solution were infused for Sham and Sch alone groups, respectively, without LPS administration.

**Table 1:** The number of animals for different experimental groups and various parameters at 90 min after LPS infusion.

Groups	Sham	Sch	LPS	Sch 0.625 + LPS	Sch 1.25 + LPS	Sch 2.5 + LPS	Sch 5 + LPS	Total
ELISA	6		6	6	6	6	6	**36**
Leukocyte adhesion	6	6	6			6		**24**
Flow cytometry	6	6	6			6		**24**
Immunohisto-chemistry	(3)	(3)	(3)			(3)		**(12)**
MPO activity assay	(5)	(5)	(5)			(5)		**(20)**
Western blotting	(5)	(5)	(5)			(5)		**(20)**
**Total**	**18**	**12**	**18**	**6**	**6**	**18**	**6**	**84**

*Sham, sham group; Sch, sch alone group; LPS, LPS infusion for 90 min group; Sch 0.625 + LPS, Sch 1.25 + LPS, Sch 2.5 + LPS, and Sch 5 + LPS represent Sch pretreatment at 0.625, 1.25, 2.5, and 5 mg/kg/h followed by LPS infusion for 90 min group, respectively. The same rat was used for assessment of flow cytometry, immunohistochemistry, MPO activity assay and Western blotting. Number in parentheses means the same rats were used as the upper row.*

In another set of experiment, rats were randomly divided into six groups (see **Table 2** for details), and anesthetized with pentobarbital sodium (60 mg/kg, i.p.). After LPS and Sch administration, rats were allowed to recover from anesthesia after treatment and freely access water and rodent chow. Eight hours after the termination of LPS infusion, rats were killed for parameter detection.

**Table 2:** The number of animals for different experimental groups and various parameters at 8 h after the termination of LPS infusion.

Groups	Sham	Sch	LPS	Sch 1.25 + LPS	Sch 2.5 + LPS	Sch 5 + LPS	Total
Evans blue extravasation	6	6	6	6	6	6	**36**
Wet-to-dry weight ratio	6	6	6	6	6	6	**36**
Histology and immunohistochemistry	(3)	(3)	(3)		(3)		**(12)**
MPO activity assay	(5)	(5)	(5)		(5)		**(20)**
Western blotting	(5)	(5)	(5)		(5)		**(20)**
Bronchoalveolar lavage fluid ELISA	6	6	6		6		**24**
**Total**	**18**	**18**	**18**	**12**	**18**	**12**	**96**

*Sham, sham group; Sch, sch alone group; LPS, LPS infusion for 90 min group; Sch 1.25 + LPS, Sch 2.5 + LPS, and Sch 5 + LPS represent Sch pretreatment at 1.25, 2.5, and 5 mg/kg/h followed by LPS infusion for 90 min group, respectively. The same rat was used for assessment of wet-to-dry weight ratio, histology and immunohistochemistry, MPO activity assay and Western blotting. Number in parentheses means the same rats were used as the upper row.*

### Observation of Leukocyte Adhesion to Lung Venules

Leukocyte adhesion to lung venules was observed 90 min after LPS infusion with an upright intravital fluorescent microscope system (BX51WT, Olympus, Tokyo, Japan), as described previously (Ma et al., 2014). In brief, the rats were placed in supine position and a thoracotomy was performed on the left chest between the third and fifth ribs to expose the left lung lobe with being ventilated with a positive pressure respirator (ALC-V8, Alcott Biotech, Shanghai, China). Observation took place approximately 30 s, during which the ventilator was turned off while the ventilation tube was infused with continuous air to make sure that the lung surface beneath a thin glass plate was expanded enough to maintain stable. The lung stayed damp by continuously dropping warm saline (37°C). For observation of the leukocyte adhering to the wall of the lung venule, the rats were administered with fluorescence tracer rhodamine 6G (1.5 mg/kg) via the femoral vein, and venules with diameter ranging from 30 to 50 μm and length of 200 μm were selected. The leukocytes that adhered to the lung venular wall for more than 10 s were defined as the adhering leukocytes.

### Assessment of EB Extravasation

Extravasation of EB dye in lungs was assessed 8 h after termination of LPS infusion. For this, EB (50 mg/kg) was injected intravenously and thoracotomy was performed 1 h thereafter. The lungs were perfused with saline via the right ventricle to rinse intravascular EB. Lungs were removed, blotted dry with tissue, weighed, cut into pieces and incubated in formamide (1 mL/100 mg tissue) for 18 h at 60°C. Optical density of the supernatant was determined by spectrophotometry at 610 nm (EB) and 740 nm (overlap of hemoglobin in the EB range). The corrected EB absorbance was calculated by the following formula: OD610-[1.426 × OD740+0.03] (Aman et al., 2012). Extravasated EB content in lung homogenates was calculated against a standard curve and expressed as μg EB per g lung tissue.

### Determination of Lung Wet-to-Dry Weight Ratio

The severity of pulmonary edema was measured by calculating the wet-to-dry weight ratio of lung tissues (Jiang et al., 2018). The animals were killed under anesthesia, and left lung lobe was excised and weighed (wet weight). The sample was then dried at 60°C for 72 h in an evaporator until a constant weight was reached (dry weight), and the wet-to-dry weight ratio was calculated by dividing the wet weight by the dry weight.

### Bronchoalveolar Lavage

To perform bronchoalveolar lavage (BAL), a 16-gauge angiocath was plugged into the rat trachea, through which 4 mL of sterile saline was instilled and then aspirated. The process was repeated three times. The resultant BAL fluid (BALF) was centrifuged at 500 *g* for 10 min, and the supernatant were collected for determination of cytokines and chemokines.

### Measurement of Cytokines, Chemokines and MPO

Rat was sacrificed following blood collection. The right inferior lung lobe was dissected and homogenized. The level of IL-1β, TNF-α, IL-6, CXCL-1 and MCP-1 in serum, lung tissue homogenate, and BALF was determined by using ELISA kits (R&D system, Minneapolis, MN, United States) according to the manufacturer’s instructions. The MPO activity of lung tissue homogenate was measured using MPO activity detection kit (Invitrogen, Rochford, IL, United States) as per the manufacturer’s instructions.

### Flow Cytometry for the Expression of CD11b and CD18 in Neutrophils

Blood was collected from the abdominal aorta and anticoagulated with heparin (20 unit/mL), followed by incubation with 1 μg FITC-labeled antibody against CD11b or CD18 (BD Biosciences Pharmingen, San Jose, CA, United States) for 20 min at room temperature. The red blood cells were lysed with hemolysin (BD Biosciences Immunocytometer Systems, San Jose, CA, United States), and the samples were washed twice with PBS. Flow cytometer (FACS Calibur, BD Biosciences, San Jose, CA, United States) was used to access the mean fluorescence intensity. For this purpose, the neutrophils were selected by FSC-SSC scattergram, and 5000 neutrophils were acquired for each sample, and the mean fluorescence intensity of each group was evaluated.

### Histological Examination

The animals were killed under anesthesia at the end of experiment, and the middle lobes of right lung were excised, rinsed in saline and fixed with 4% paraformaldehyde in 0.01 M PBS (pH 7.4). The tissues were cut into blocks, embedded in paraffin, and sectioned to 5 μm sections. The sections were stained by hematoxylin and eosin (HE). The images of lung tissue were captured by a digital camera connected to a microscope (BX512DP70, Olympus, Tokyo, Japan).

### Immunohistochemistry

The lung tissue sections were deparaffinized by xylene and graded ethanol. Endogenous peroxidase was blocked by incubating in 3% H_2_O_2_-methanol at room temperature for 30 min. After washing in phosphate buffered saline (PBS), slides were incubated with 5% normal goat serum in PBS at 37°C for 30 min to prevent non-specific staining. The slides were then incubated with antibody directed to MPO (1: 100) diluted in PBS containing 1% bovine serum albumin overnight at 4°C. Specific binding was detected by incubation with horseradish peroxidase (HRP)-conjugated secondary antibody (ZSGB-BIO, Beijing, China) and revealed with the 3,3′-diaminobenzidine (DAB) substrate Kit. The images were captured by a digital camera connected to a microscope (BX512DP70, Olympus, Tokyo, Japan).

### Immunofluorescence Staining and Confocal Microscopy

Sections were deparaffinized and treated with 0.01 M sodium citrate (pH 6.0) for antigen retrieval, washed by PBS, and permeabilized with 0.3% Triton X-100 for 30 min. Following blocking with goat serum at room temperature for 30 min, sections were incubated with primary antibodies against Claudin-5 (1: 50), vWF (1: 50) and keratin (1: 50) diluted in PBS overnight at 4°C, respectively. After being rinsed with PBS, sections were incubated with Dylight488-labeled secondary antibodies (1: 100, KPL, Gaithersburg, MD, United States) for 2 h at 37°C. TUNEL staining was undertaken by a cell death detection kit (Roche, Basel, Switzerland), according to the manufacture’s instruction. All of the sections were counterstained with Hoechst 33342 for nuclei. Images were acquired using a laser scanning confocal microscope (TCS SP8, Leica, Mannheim, Germany).

### Western Blotting

The right inferior lung lobe was harvested at the time point indicated, and lung tissues were homogenized in lysis buffer (Applygen Technologies, Beijing, China) containing the protease inhibitor. Nuclear protein was extracted by NE-PER nuclear and cytoplasmic extraction reagents kit (Thermo Scientific, Kansas, MA, United States) according to the manufacturer’s instruction. The concentration of whole protein and nuclear protein was determined with a BCA protein assay kit (Applygen Technologies, Beijing, China). The proteins were separated on SDS-PAGE, and transferred to polyvinylidene difloride membrane. Following blocking and rinsing with TBS-Tween (TBST), the membrane with target proteins was cut and incubated overnight at 4°C with antibodies, respectively, against I-κBα (1: 2000), phospho-I-κBα (1: 1000), NF-κB p65 (1: 1000), Erk (1: 2000), phospho-Erk1/2 (1: 1000), p38 MAPK (1: 2000), phospho-p38 MAPK (1: 1000), TLR4 (1: 400), VE-Cadherin (1: 300), Claudin-5 (1: 400), Occludin (1: 400), ZO-1 (1: 1000), FoxO1 (1: 2000), phospho-FoxO1 (1: 1000), Akt (1: 2000), phospho-Akt (1: 1000), Keratin (1: 1000), vWF (1: 1000), proSP-C (1: 1000), caspase-3 (1: 500), p27^kip1^ (1: 1000), p21^cip1^ (1: 500), cyclin D1 (1: 1000), phospho-Rb (1: 1000), and cyclin B1 (1: 1000). The GAPDH (1: 5000), and histone H3 (1: 1000) were applied as loading control for whole and nucleus protein, respectively. After rinsing with TBST, the membranes were incubated with secondary antibody (Cell Signaling Technology, Beverly, MA, United States) for 1 h at room temperature. The blots were developed using the SuperEnhanced Chemiluminescence detection kit (Applygen Technologies Inc., Beijing, China), and the protein bands were visualized after exposure of the membranes to Kodak X-ray film. The intensity of each band was evaluated with Quantity One software (Bio-Rad, Hercules, CA, USA). For statistical analysis, the intensity ratio of target protein in each group to loading control in the same lane was calculated and presented as the fold change over Sham group.

### Statistical Analysis

All parameters were expressed as means ± SD. Differences between three or more groups were assessed using one-way ANOVA with a Bonferroni multiple comparisons test. Values of *p* < 0.05 were considered to be significant.

## Results

### Sch Inhibits LPS-Induced Systemic and Lung Inflammation in a Dose-Dependent Manner

**FIGURE 2:**
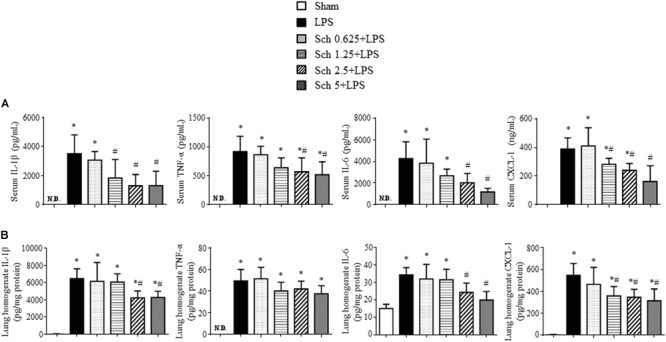
Sch reduces the levels of proinflammatory cytokines and chemokine in serum and lung tissues 90 min after LPS infusion. **(A)** ELISA for IL-1β, TNF-α, IL-6, and CXCL-1 in the serum in different groups. **(B)** ELISA for IL-1β, TNF-α, IL-6, and CXCL-1 in the lung tissues in different groups. N.D., not detected. Sham, sham group; LPS, LPS infusion for 90 min group; Sch 0.625 + LPS, Sch 1.25 + LPS, Sch 2.5 + LPS, and Sch 5 + LPS represent Sch pretreatment at 0.625, 1.25, 2.5, and 5 mg/kg/h followed by LPS infusion for 90 min group, respectively. Values are expressed as mean ± SD of 6 separate experiments. ^∗^*p* < 0.05 vs. Sham group, ^#^*p* < 0.05 vs. LPS group.

To evaluate the role of Sch in the inflammatory response to LPS stimulation, we analyzed the serum levels of the pro-inflammatory cytokines IL-1β, TNF-α and IL-6, as well as chemokine CXCL-1 90 min after LPS infusion. The serum levels of IL-1β, TNF-α, IL-6, and CXCL-1 elevated markedly after LPS stimulation compared with those in Sham rats. In contrast, Sch pretreatment dose-dependently inhibited the increase in serum levels of cytokines and chemokine caused by LPS (**Figure 2A**). Pretreatment with Sch at the dose of 1.25, 2.5, and 5 mg/kg/h all significantly inhibited the LPS-induced serum cytokines and chemokine production, with 1.25 mg/kg/h being less efficient than the other two, while Sch at the dose of 0.625 mg/kg/h had no effect (**Figure 2A**). Similarly, the levels of IL-1β, IL-6, and CXCL-1, but not TNF-α, in lung tissue were significantly decreased by Sch pretreatment in a dose-dependent manner compared with LPS group, being significant from 1.25 mg/kg/h as well (**Figure 2B**). So we chose the mid-dose (2.5 mg/kg/h) for the following experiments.

### Sch Reduces the Number of Leukocytes Adherent to Lung Venules and Attenuates the Upregulation of Adhesion Molecules

**FIGURE 3:**
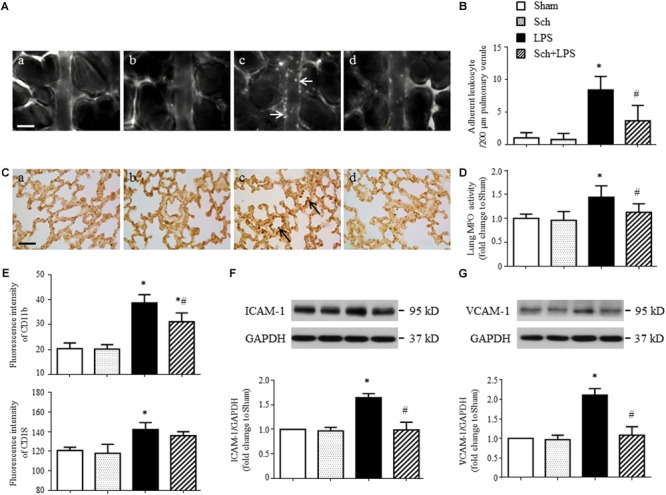
Sch inhibits LPS-induced leukocyte adhesion to the pulmonary venules and the expression of adhesion molecules 90 min after LPS infusion. **(A)** Representative images showing leukocyte adhesion to the pulmonary venular wall in Sham **(a)**, Sch alone **(b)**, 90 min after LPS infusion **(c)**, and Sch pretreatment at 2.5 mg/kg/h followed by LPS infusion for 90 min **(d)** group. Arrows indicate adherent leukocytes. Bar = 50 μm. **(B)** Statistical result of the number of leukocytes adherent to the pulmonary venules (*N* = 6). **(C)** Representative images of immunohistochemical staining for MPO in Sham **(a)**, Sch alone **(b)**, 90 min after LPS instillation **(c)**, and Sch pretreatment at 2.5 mg/kg/h followed by LPS infusion for 90 min **(d)** group. All images are representative of 3 separate experiments. Arrows indicate recruited leukocytes in pulmonary venules. Bar = 50 μm. **(D)** Quantification of MPO activity in rat lung tissues in different groups (*N* = 5). **(E)** The expression of CD11b and CD18 on neutrophils measured by flow cytometry (*N* = 6). **(F,G)** The expression of ICAM-1 **(F)** and VCAM-1 **(G)** in the lung tissue assessed by Western blotting (*N* = 5). Sham, sham group; Sch, sch alone group; LPS, LPS infusion for 90 min group; Sch + LPS, Sch pretreatment at 2.5 mg/kg/h followed by LPS infusion for 90 min group. Values are expressed as mean ± SD. ^∗^*p* < 0.05 vs. Sham group, ^#^*p* < 0.05 vs. LPS group.

To examine the effect of Sch on the lung microcirculatory disturbances induced by LPS, the leukocyte adhesion to lung venules was observed with an upright intravital fluorescent microscope. Shown in **Figure 3A** are the representative images of lung venules from different groups, in which there was no leukocyte adhesion to the lung venules in Sham (**Figure 3A-a**) and Sch alone (**Figure 3A-b**) group, while a considerable leukocytes adhering to the lung venules was observed in LPS group (**Figure 3A-c**). Obviously, pretreatment with Sch reduced the number of leukocytes adhering to the lung venules induced by LPS (**Figure 3A-d**). The quantitative evaluation of the results (**Figure 3B**) confirmed the view from image inspection. The protective effect of Sch on the accumulation of leukocytes in lung venules after LPS stimulation was further proved by MPO immunohistochemical staining and MPO activity assay (**Figures 3C,D**). Adhesion molecules CD11b/CD18 (Mac-1), ICAM-1, and VCAM-1 are considered as the mediators for leukocyte adhesion to inflamed endothelium, thus the expression of CD11b/CD18 on neutrophils and ICAM-1/VCAM-1 in lung tissues were determined by flow cytometry and Western blotting, respectively. As expected, the expression of CD11b/CD18 and ICAM-1/VCAM-1 increased 90 min after LPS infusion, all of which but CD18 were inhibited by Sch pretreatment (**Figures 3E–G**).

### Sch Decreases the LPS-Induced Expression of TLR-4 and Activation of NF-κB and MAPK

**FIGURE 4:**
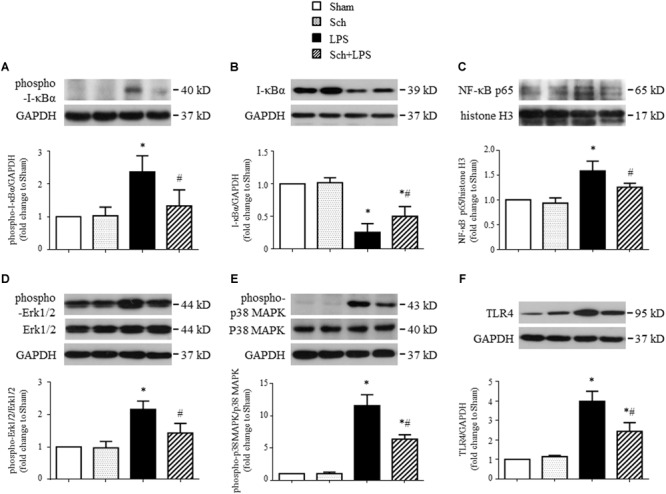
Sch inhibits the expression of TLR-4 in lung tissues and activation of NF-κB and MAPK signaling pathway 90 min after LPS infusion. **(A,B)** The phosphorylation **(A)** and degradation **(B)** of IκB-α. **(C)** The nuclear expression of NF-κB p65 subunit. **(D–F)** The phosphorylation of Erk1/2 **(D)** and p38 MAPK **(E)**, and expression of TLR-4 **(F)**. Sham, sham group; Sch, sch alone group; LPS, LPS infusion for 90 min group; Sch + LPS, Sch pretreatment at 2.5 mg/kg/h followed by LPS infusion for 90 min group. Values are expressed as mean ± SD of 5 separate experiments. ^∗^*p* < 0.05 vs. Sham group, ^#^*p* < 0.05 vs. LPS group.

To gain insights into the signaling implicated in the inhibition effects of Sch on inflammatory reaction, the expression of TLR-4 and activation of NF-κB and MAPK in lung tissues were assessed in different conditions. As shown in **Figure 4**, LPS infusion for 90 min resulted in a sharp increase in the I-κBα phosphorylation (**Figure 4A**) and a decrease in I-κBα expression (**Figure 4B**), which, as expected, resulted in NF-κB p65 nuclear translocation (**Figure 4C**), indicating the activation of NF-κB signaling pathway. Pretreatment with Sch significantly inhibited the activation of NF-κB signaling pathway (**Figures 4A–C**). Moreover, Sch pretreatment significantly attenuated the phosphorylation of Erk1/2 and p38 MAPK induced by LPS (**Figures 4D,E**), indicating an involvement of MAPK signaling pathway in Sch action. Moreover, TLR-4, serving as the receptor of LPS and upstream of NF-κB and MAPK, increased 90 min after LPS infusion, which was consistent with previous studies (Li et al., 2013; Zhang et al., 2016), also inhibited by Sch pretreatment (**Figure 4F**).

### Sch Attenuates LPS-Induced Lung Microvessel Hyperpermeability

**FIGURE 5:**
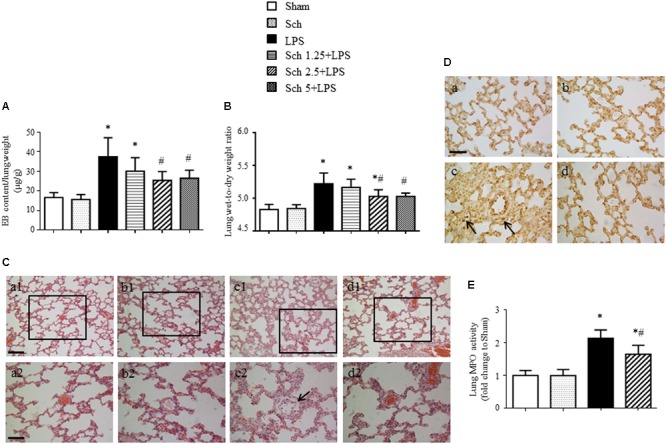
Sch alleviates pulmonary microvascular hyperpermeability at 8 h after the termination of LPS infusion. **(A)** Evens blue extravasation at 8 h after the termination of LPS infusion (*N* = 6). **(B)** Lung wet-to-dry weight ratio at 8 h after the termination of LPS infusion (*N* = 6). **(C)** Representative images of HE staining in Sham **(a)**, Sch alone **(b)**, 8 h after the termination of LPS infusion **(c)**, and 8 h after the termination of LPS infusion in the presence of Sch pretreatment (2.5 mg/kg/h) **(d)** group. Bar = 100 μm **(a1–d1)** and 50 μm **(a2–d2)**. **(D)** Representative images of MPO immunohistochemical staining in Sham **(a)**, Sch alone **(b)**, 8 h after the termination of LPS infusion **(c)**, and 8 h after the termination of LPS infusion in the presence of Sch pretreatment (2.5 mg/kg/h) **(d)** group. Bar = 50 μm. All images are representative of 3 separate experiments. Arrows indicates perivascular edema **(C)** and infiltration of leukocyte in pulmonary tissues **(D)**. **(E)** Quantification of MPO activity in rat lung tissues in different groups (*N* = 5). Sham, sham group; Sch, sch alone group; LPS, LPS infusion for 90 min group; Sch 1.25 + LPS, Sch 2.5 + LPS and Sch 5 + LPS represent Sch pretreatment at 1.25, 2.5, and 5 mg/kg/h followed by LPS infusion for 90 min group, respectively. Values are expressed as mean ± SD. ^∗^*p* < 0.05 vs. Sham group, ^#^*p* < 0.05 vs. LPS group.

To further assess the development of lung microvessel hyperpermeability after LPS stimulation, we measured lung EB extravasation and wet-to-dry weight ratio at 8 h after the termination of LPS and revealed a reduction in transvascular flux of EB and lung wet-to-dry weight ratio in the presence of Sch, with a dose-dependent manner (**Figures 5A,B**). Consistent with these results, compared to Sham and Sch alone group (**Figures 5C-a,b** and **Figures 5D-a,b**), histology study performed 8 h after termination of LPS infusion showed an obvious edema around microvessels (**Figure 5C-c**, arrow), thickened interstitium and leukocyte accumulation within the interstitium (**Figure 5D-c**, arrow). In addition, lung tissue from LPS group showed a significantly higher MPO activity relative to Sham and Sch alone groups (**Figure 5E**). Excitingly, both histological abnormalities and leukocyte infiltration of lung tissue after LPS were attenuated by Sch pretreatment evidently (**Figures 5C-d,D-d,E**), indicating the protection effects of Sch on microvascular hyperpermeability during LPS stimulation.

### Sch Promotes Endothelial Regeneration Following LPS Induced Vascular Injury

**FIGURE 6:**
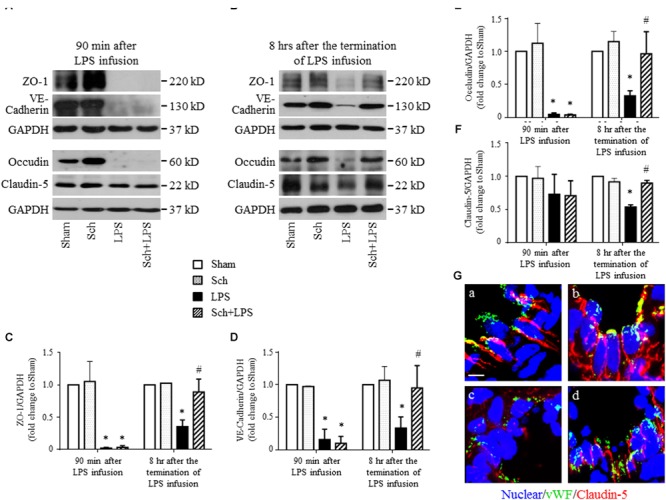
Sch recovers the expression of endothelial tight junction and adherent junction protein at 8 h after the termination of LPS infusion. **(A,B)** Representative Western blotting images of ZO-1, VE-Cadherin, Occludin, and Claudin-5 at 90 min after LPS infusion **(A)**, and 8 h after the termination of LPS infusion **(B)**, respectively, among different groups. **(C–F)** Quantitative analysis of ZO-1 **(C)**, VE-Cadherin **(D)**, Occludin **(E)**, and Claudin-5 **(F)** at each time point after the LPS administration in different groups. Values are expressed as mean ± SD of 5 separate experiments. **(G)** Representative immunofluorescence confocal images for Claudin-5 in Sham **(a)**, Sch alone **(b)**, 8 h after the termination of LPS infusion **(c)**, and 8 h after the termination of LPS infusion in the presence of Sch pretreatment (2.5 mg/kg/h) **(d)** group. All images are representative of 3 separate experiments. Bar = 5 μm. Sham, sham group; Sch, sch alone group; LPS, LPS infusion for 90 min group; Sch + LPS, Sch pretreatment at 2.5 mg/kg/h followed by LPS infusion for 90 min group. ^∗^*p* < 0.05 vs. Sham group, ^#^*p* < 0.05 vs. LPS group.

**FIGURE 7:**
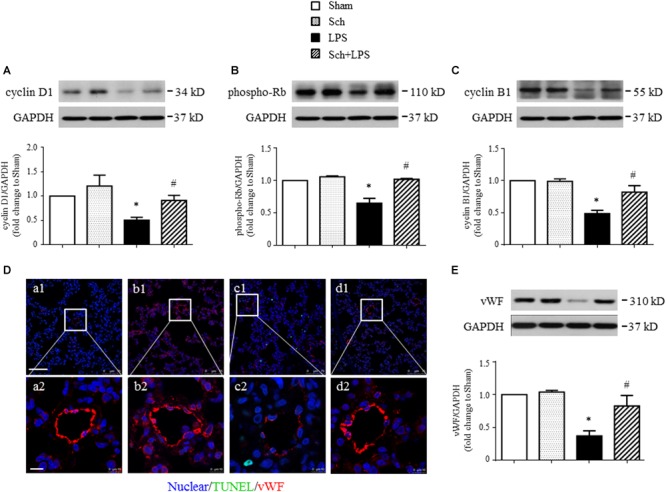
Sch promotes endothelial cell regeneration at 8 h after the termination of LPS infusion. **(A–C)** Representative Western blots and quantitative analysis of cyclin D1 expression **(A)**, Rb phosphorylation **(B)**, and cyclin B1 expression **(C)** in different groups. Values are expressed as mean ± SD of 5 separate experiments. **(D)** Representative immunofluorescence confocal images for vWF in Sham **(a)**, Sch alone **(b)**, 8 h after the termination of LPS infusion **(c)**, and 8 h after the termination of LPS infusion in the presence of Sch pretreatment (2.5 mg/kg/h) **(d)** group. All images are representative of 3 separate experiments. Bar = 75 μm **(a1–d1)** and 10 μm **(a2–d2)**. **(E)** Quantitative analysis of vWF in different groups. Values are expressed as mean ± SD of 5 separate experiments. Sham, sham group; Sch, sch alone group; LPS, LPS infusion for 90 min group; Sch + LPS, Sch pretreatment at 2.5 mg/kg/h followed by LPS infusion for 90 min group. ^∗^*p* < 0.05 vs. Sham group, ^#^*p* < 0.05 vs. LPS group.

We next explored the rationale for the microvascular protective potential of Sch. For this purpose, the expression of the three major tight junction proteins Claudin-5, Occludin, ZO-1, together with adherent junction protein VE-Cadherin was assessed by Western blotting and confocal microscopy. The result showed that LPS infusion for 90 min caused a significant decrease in the expression of both tight junction proteins (although not significant for Claudin-5) and adherent junction protein compared to Sham and Sch alone groups (**Figure 6A**), which sustained until 8 h after the termination of LPS stimulation (**Figure 6B**). Sch pretreatment had no effect on the LPS-reduced expression of junction proteins 90 min after LPS infusion (**Figures 6A,C–F**), which, however, recovered the expression of junction proteins at 8 h after termination of LPS stimulation (**Figures 6B,C–F**). The role of Sch in recovery of Claudin-5 at 8 h after LPS administration was verified by confocal microscopy (**Figure 6G**), revealing a decreased expression and a discontinuous distribution of Claudin-5 in lung tissues 8 h after the LPS administration, which was obviously recovered by treatment with Sch. These results suggested that LPS-injured endothelial cells underwent a regeneration process in the presence of Sch. To validate this inference, we determined by Western blotting the expression of major proteins essential for cell cycle progression, including cyclin D1 and phosphorylated Rb required for G1/S transition, and cyclin B1 for G2/M transition. The results showed that these proteins were down-regulated in lungs at 8 h after the LPS administration, all of which were reversed by Sch pretreatment significantly (**Figures 7A–C**). The endothelial regeneration in the presence of Sch was further confirmed by the expression of endothelial marker, vWF, as shown by both confocal microscopy (**Figure 7D**) and Western blotting (**Figure 7E**).

### Sch Inhibits Pulmonary Epithelial Cell Apoptosis

**FIGURE 8:**
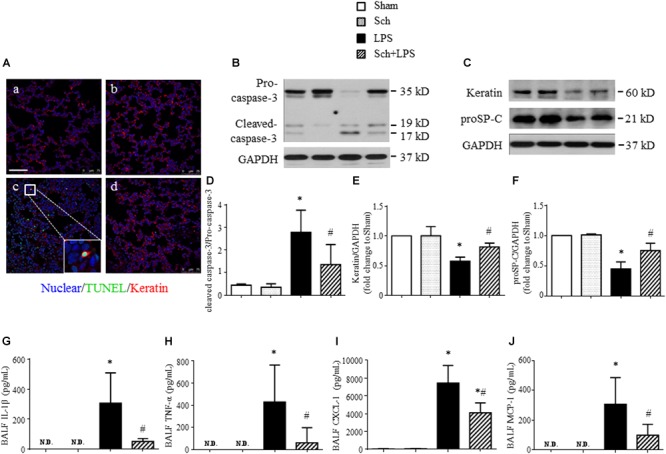
Sch abrogates epithelial apoptosis at 8 h after the termination of LPS infusion. **(A)** Representative immunofluorescence confocal images for double staining with TUNEL and keratin in Sham **(a)**, Sch alone **(b)**, 8 h after the termination of LPS infusion **(c)**, and 8 h after the termination of LPS infusion in the presence of Sch pretreatment (2.5 mg/kg/h) **(d)** group. All images are representative of 3 separate experiments. Inserted image indicates colocalization of TUNEL and kerarin positive cell. Bar = 75 μm. **(B)** Representative Western blots of caspase-3 in different groups. **(C)** Representative Western blots of Keratin and proSP-C in different groups. **(D–F)** Quantification analysis of caspase-3 **(D)**, keratin **(E)**, and proSP-C **(F)**. Values are expressed as mean ± SD of 5 separate experiments. **(G–J)** ELISA assay for IL-1β, TNF-α, CXCL-1, and MCP-1 in BALF in different groups. N.D., not detected. Values are expressed as mean ± SD of 6 separate experiments. Sham, sham group; Sch, sch alone group; LPS, LPS infusion for 90 min group; Sch + LPS, Sch pretreatment at 2.5 mg/kg/h followed by LPS infusion for 90 min group. ^∗^*p* < 0.05 vs. Sham group, ^#^*p* < 0.05 vs. LPS group.

In addition to microvessels, LPS challenge may impair alveolar epithelial cells manifesting apoptosis. We thus explored if Sch had effect on LPS-injured alveolar epithelial cells. Examinations undertook at 8 h after the termination of LPS infusion revealed an increased apoptosis and a decreased expression of keratin in lung tissue, as shown by Western blotting (**Figures 8B,C**). The localization of TUNEL-positive cells and keratin in confocal microscope image showed that most of apoptosis occurred in epithelial cells (**Figure 8A-c**, inserted), compared to Sham (**Figure 8A-a**), Sch alone (**Figure 8A-b**), and Sch pretreatment group (**Figure 8A-d**). Sch pretreatment significantly blunted the LPS-induced lung epithelial cells injury, as evidenced by the decrease in cleaved caspase-3 and increase in keratin and proSP-C (a specific marker of type II epithelial cell) (**Figures 8B–F**).

### Sch Reduces Inflammatory Mediators in BALF After LPS

Lipopolysaccharide stimulation activates leukocytes, which migrate into alveolar spaces and release inflammatory mediators resulting in apoptosis and necrosis of alveolar epithelial type I and type II cells (Zemans et al., 2009). As expected, a profound increased expression of cytokines, including IL-1β and TNF-α, as well as chemokines, such as CXCL-1 and MCP-1 was detected in BALF at 8 h after the termination of LPS infusion (**Figures 8G–J**). Sch pretreatment markedly reduced the level of the cytokines and chemokines in BALF after LPS (**Figures 8G–J**), suggesting that Sch protected lung epithelial cells from apoptosis may be attributed to prevent leukocytes migration.

### Sch Modulates Endothelial and Epithelial Regeneration via Regulating FoxO1 Function

**FIGURE 9:**
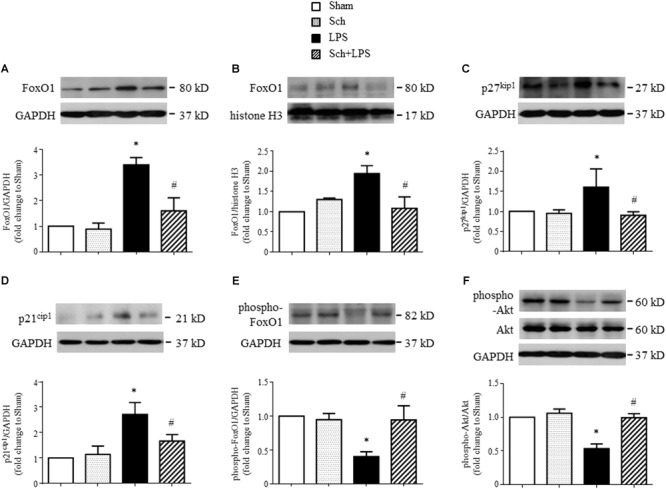
Sch inhibits FoxO1 activation at 8 h after the termination of LPS infusion. **(A,B)** Representative Western blots and quantitative analysis of FoxO1 expression in cytoplasm **(A)** and nuclei **(B)** in different groups. **(C,D)** Representative Western blots and quantitative analysis of p27^kip1^ **(C)** and p21^cip1^ **(D)** in different groups. **(E,F)** Representative Western blots and quantitative analysis of FoxO1 **(E)** and Akt **(F)** phosphorylation in different groups. Values are expressed as mean ± SD of 5 separate experiments. Sham, sham group; Sch, sch alone group; LPS, LPS infusion for 90 min group; Sch + LPS, Sch pretreatment at 2.5 mg/kg/h followed by LPS infusion for 90 min group. ^∗^*p* < 0.05 vs. Sham group, ^#^*p* < 0.05 vs. LPS group.

FoxO1 is a key transcription factor involved in pro-cell-cycle arrest process (Eijkelenboom and Burgering, 2013), we next explored by Western blotting whether FoxO1 plays a role in Sch protective effects. The results revealed an upregulated expression (**Figure 9A**) and increased nuclear translocation (**Figure 9B**) of FoxO1 at 8 h after the termination of LPS infusion, suggesting that FoxO1 may function in nucleus to regulate gene transcription. This notion was supported by the increase in p27^kip1^ and p21^cip1^, two of the major cyclin dependent kinase inhibitors (**Figures 9C,D**), which serve as the target protein of FoxO1 (van der Vos and Coffer, 2011). Importantly, all the alterations after LPS were abrogated by Sch pretreatment (**Figures 9A–D**). Because phosphorylation is commonly associated with FoxO1 nuclear exclusion and degradation, we next assessed the phosphorylation of FoxO1 in different conditions, and found a decrease in FoxO1 phosphorylation upon LPS stimulation, which was up-regulated by Sch (**Figure 9E**). FoxO1 transcriptional activity is regulated by a variety of mechanisms including activation of Akt, which directly phosphorylates FoxO1 leading to nuclear export and inactivation (Brunet et al., 1999). As expected, a remarkable decrease of Akt phosphorylation was observed at 8 h after the termination of LPS infusion, as compared to Sham, while Sch significantly reversed the down-regulated Akt phosphorylation (**Figure 9F**). Taking together, these data suggested that Akt/FoxO1 signaling regulation is implicated in the preventive role of Sch.

## Discussion

Acute lung injury results from a diversity of risk factors including Gram-negative sepsis, while has a pathology in common in acute phase manifesting alveolar flooding with protein rich fluid secondary to a loss of integrity of the normal alveolar capillary base. Consistent with this, we observed at 8 h after 90 min LPS infusion a lung microvascular hyperpermeability and lung edema, along with a decreased endothelial and epithelial cells markers vWF and keratin, and an increased pro-inflammatory cytokine in BALF. Importantly, all the alterations were significantly protected by Sch treatment, suggesting Sch as a promising alternative to deal with the development of ALI.

Acute lung injury occurs as a pathological process initiating with inflammatory reaction characterized by a burst of inflammatory mediators and recruitment of leukocytes in microvessels (Abraham, 2003). Following LPS infusion for 90 min, the pro-inflammatory cytokines and chemokines increased in both serum and lung tissue, while leukocytes were found recruited in lung venules with an elevated expression of related adhesion molecules, indicating the occurrence of lung inflammation. Moreover, increased expression of TLR-4 and activation of NF-κB/MAPK confirmed the occurrence of inflammation and involvement of this signaling pathway. Most of the changes were attenuated by Sch, indicating that the preventive role of this drug for ALI started in the early phase of the pathogenesis and involved interfering in TLR-4/NF-κB/MAPK signaling.

Accumulation of neutrophils in lungs and the production of inflammatory mediators are a crucial episode in the progressing of ALI. Among the cytokines evaluated in the present study, TNF-α is particularly worth motioning. TNF-α is known to dysregulate tight junction proteins and actin arrangement leading to a disruption of endothelial and epithelial barrier (Capaldo and Nusrat, 2009). In addition, TNF-α is also shown to induce apoptosis in many cell types including endothelial and epithelial cells (Sprague and Khalil, 2009). In agreement with the recognized effect of TNF-α, we observed a significantly increased TNF-α in serum and lung tissue immediately after LPS infusion for 90 min, as well as in BALF at 8 h after the termination of LPS infusion, which was in concurrence of decreased expression of tight junction and adherent junction proteins and increased EB extravasation and lung tissue wet-to-dry weight ratio. Of notice, after LPS infusion for 90 min, Sch had no attenuating effect on increased TNF-α in lung tissue, nor on decreased expression of junction proteins. Whereas, Sch attenuated the increased TNF-α in BALF, as well as the increased EB extravasation and lung tissue wet-to-dry weight ratio at 8 h after the termination of LPS infusion. These results not only verified the critical importance of TNF-α in regulation of endothelial and epithelial barrier, but also shed light on the mechanism thereby Sch maintains barrier integrity.

Apoptosis and necrosis of endothelial and epithelial cells are known to contribute to ALI and ARDS, resulting in flooding of airspaces and breakdown in the gas exchange (Ware and Matthay, 2001; Albertine et al., 2002; Matthay et al., 2012; Gill et al., 2015). Promoting regeneration of the injured endothelial and epithelial cells is critical for ALI progression from acute phase to resolution phase. The role of Sch in helping injured endothelial cells regeneration exhibited as the recovery of vWF expression in the presence of Sch, which together with the recovered expression of junction proteins contributes to restoration of endothelium barrier. Injury to type I alveolar epithelial cells causes the collapse of alveolar epithelial integrity, while injury to type II alveolar epithelial cells reduces surfactant production and impairs the removal of edematous fluid from the alveolar space. As expected, we found an increased apoptosis and a decreased keratin and proSP-C expression after LPS infusion, suggestive of damage to alveolar epithelial cells. Sch attenuated the damage to alveolar epithelial cells, as evidenced by the reduced caspase-3 activation and recovery of keratin and proSP-C expression. The pro-inflammatory cytokines participate in initiating apoptosis and necrosis either directly or through induction of reactive oxygen species. It is thus likely that the role of Sch in attenuating damage to alveolar epithelial cells is at least partly accounted for by its potential to inhibit the production of pro-inflammatory cytokines. Furthermore, the expression of cyclin D1, phospho-Rb and cyclin B1, major proteins required for cell cycle progression, showed a significant decrease after LPS infusion, indicating occurrence of a dysregulated regeneration of damaged endothelial and epithelial cells. While Sch abolished this decrease, suggesting its role in regeneration of endothelial and alveolar epithelial cells, which is contributable to the preventive effect of this drug for ALI.

To gain insight into the mechanisms underlying the desirable effects of Sch on endothelial and alveolar epithelial cell regeneration, we detected the changes of transcription factor FoxO1 after LPS stimulation and the effect of Sch. Previous study has shown that FoxO transcription factors can inhibit cell cycle activators including the D-type cyclins in established cell lines (Schmidt et al., 2002). Moreover, in mice lacking p27^kip1^, a target gene of FoxO1, the cardiac cell cycle is prolonged and proliferation is extended (Poolman et al., 1999). Another report has shown that the regulation of p21^cip1^ expression by FOXO1 plays an important role in the proliferation of adipocytes. In these cells, insulin signaling repressed the FOXO1 activation and accompanied upregulation of p21^cip1^ expression, resulting in increased proliferation (Nakae et al., 2003). Consistent with these findings, the present study revealed that at 8 h after the termination of LPS infusion, both FoxO1 expression and nuclear accumulation increased, which accompanied with a down-regulated cyclin D1 and cyclin B1 expression and Rb phosphorylation, and an upregulated cyclin dependent kinase inhibitors p27^kip1^ and p21^cip1^, indicating a disturbed cell regeneration after LPS stimulation. Noticeably, all of the aforementioned changes can be reversed by Sch treatment, suggesting involvement of FoxO1 in the effect of Sch on endothelial and epithelial cell regeneration. Further study revealed an increase in FoxO1 phosphorylation and Akt activation in the presence of Sch, suggesting that Sch promoted cell cycle by inhibition of FoxO1, potentially through Akt-mediated phosphorylation and nuclear exclusion.

## Conclusion

This study demonstrated that pretreatment with Sch attenuated LPS-elicited inflammation and damage to endothelial and epithelial barrier, through mechanisms involving TLR-4/NF-κB/MAPK and Akt/FoxO1 signaling pathways. This result suggests Sch as a potential management for ALI.

## Author Contributions

J-YH and XW designed the research, obtained the funding, and led and supervised the study. KS, RH, LY, D-TL, Y-YL, X-HW, Y-CC, and C-SP conducted the experiments. KS, J-YF, and J-YH analyzed and interpreted the data. KS drafted the first version of the manuscript. KS, J-YF, and J-YH edited the manuscript. All authors approved the final version.

## Conflict of Interest Statement

The authors declare that the research was conducted in the absence of any commercial or financial relationships that could be construed as a potential conflict of interest.
